# Explantation of a sutureless scleral fixated Carlevale intraocular lens due to calcification: a clinical and laboratory report

**DOI:** 10.1186/s12886-023-03102-0

**Published:** 2023-08-16

**Authors:** Panos S. Gartaganis, Panagiota D. Natsi, Sotirios P. Gartaganis, Petros G. Koutsoukos, Evangelos Manousakis, Efthymios Karmiris

**Affiliations:** 1 Department of Ophthalmology, 251 Hellenic Air Force General Hospital, 16 Avras Str, 166 73 Athens, Greece; 2 Department of Chemical Engineering, Laboratory of Inorganic and Analytical Chemistry, University of Patras and FORTH/ICE-HT, Patras, Greece; 3 Department of Ophthalmology, Medical School, University of Patras, Patras, Greece

**Keywords:** Hydrophilic acrylic intraocular lens, Calcification, Opacification

## Abstract

**Background:**

Hydrophilic intraocular lens opacification is a rare complication due to calcification. With current new surgical techniques, including lamellar endothelial keratoplasty and vitrectomies, this irreversible complication is becoming more common. In this case study, we present clinical and laboratory features of a case of Carlevale hydrophilic acrylic IOL calcification.

**Case presentation:**

Observational case report of a single incident case. An 83-year-old man was referred to our ophthalmic department complaining of right eye vision blurring for six months. Slit-lamp biomicroscopy revealed IOL opacification. Deposits of calcium phosphate were found both on the IOL’s surface and inside it, according to thorough investigation using optical, scanning electron microscopy (SEM), and energy-dispersive X-ray (EDX) spectrometry.

**Conclusions:**

To the best of our knowledge, this is the first case to describe the laboratory evidence of Carlevale hydrophilic IOL calcification, suggesting possible explanation mechanisms based on underlying pathology and surgical technique. It reminds us that these findings suggest that physicians should be aware of possible hydrophilic IOL calcification.

**Supplementary Information:**

The online version contains supplementary material available at 10.1186/s12886-023-03102-0.

## Background

The Carlevale (FIL SSF, Soleko IOL Division, Italy) intraocular lens (IOL) is a foldable one-piece posterior chamber IOL, made of 25% water content hydrophilic acrylic material, specifically designed for sutureless scleral fixation (SSF) with two T-shaped haptics [1] The sutureless nature and design of Carlevale IOL (FIL SSF, Soleko IOL Division, Italy) has increased its popularity among surgeons in aphakic patients or in the absence of capsular support [2].

Calcification of hydrophilic IOLs remains an uncommon long-term complication of this type of IOLs. Risk of IOL calcification may be related to surgical technique in pseudophakic eyes, systemic diseases, process of IOL manufacturing and storage and perhaps most importantly the material of the IOL [2–5] Calcification is due to precipitation of calcium phosphate salts on the surface of or within the lens material and is well documented for hydrophilic IOLs. This could result in serious vision impairment, necessitating IOL exchange [6–8] In this report, we present for the first time the clinicopathologic features of hydrophilic acrylic Carlevale IOL (FIL SSF, Soleko IOL Division, Italy), which was explanted due to calcification.

### Case presentation

**Fig. 1:**
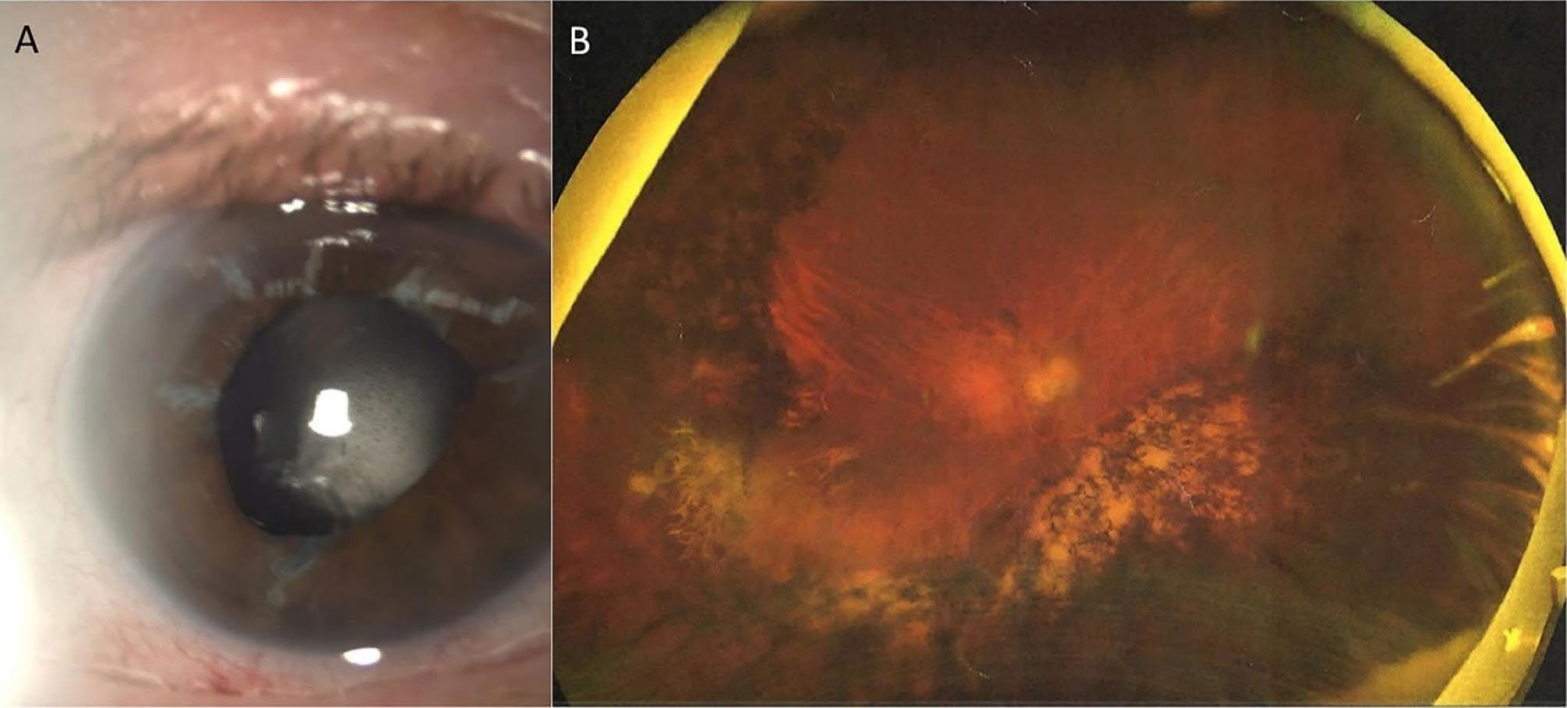
Findings at initial presentation (**A**) Slit-lamp photograph of the opacified lens. (**B**) Fundus photograph at the initial examination, the detailed vascular mapping by Optos P200DTx (Optos PLC, Dunfermline, Scotland, UK) was difficult due to opaque lens

**Fig. 2:**
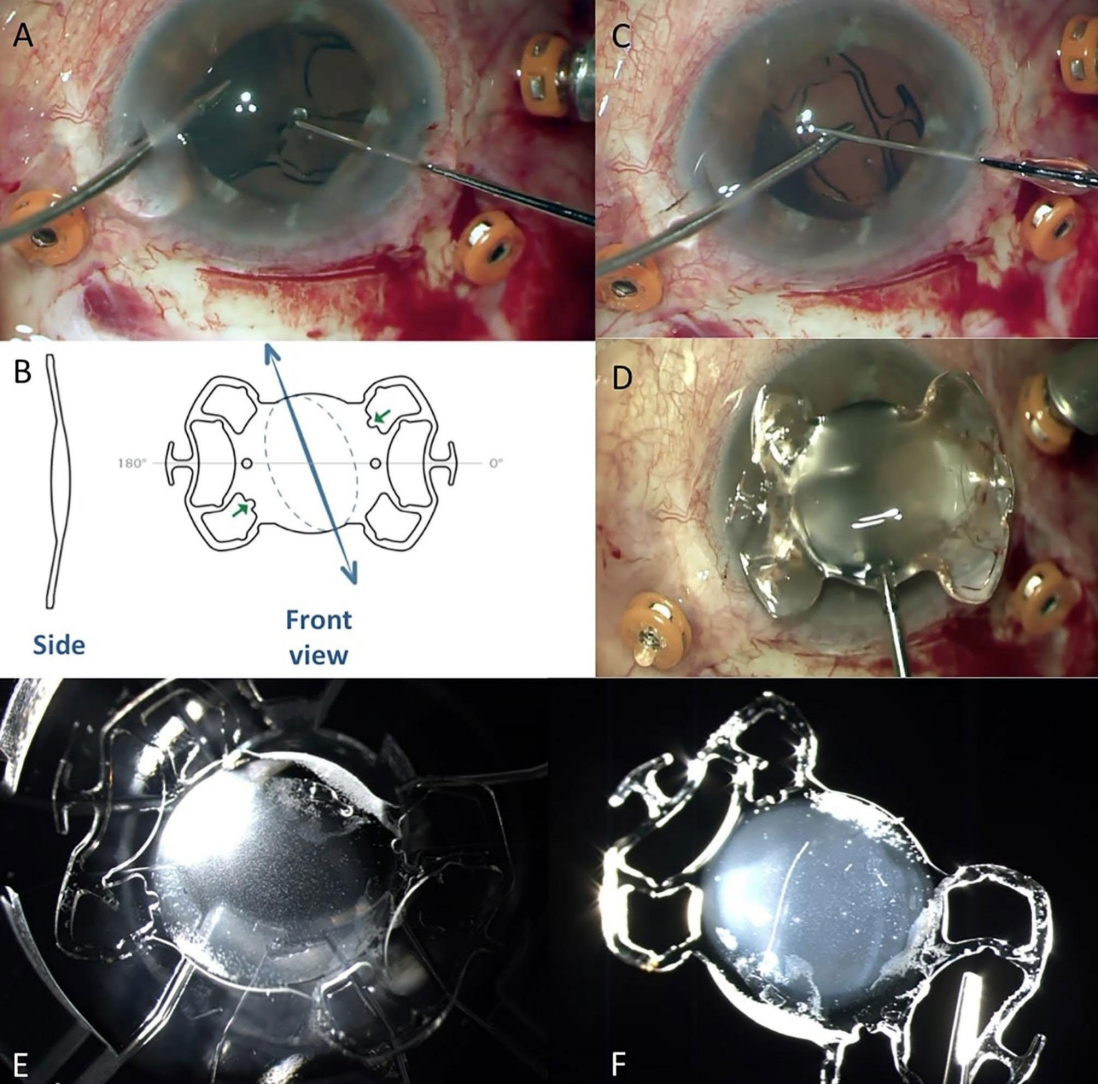
(**A**) Photograph taken during the explantation of the IOL demonstrates the reverse implantation as indicated by the brochure photograph (**B**). (**C**) Disengagement of the temporal T-shaped haptic. (**D**) Photograph of the explanted Carlevale IOL (FIL SSF, Soleko IOL Division, Italy). (**E**) Anterior surface of the IOL showing more deposits than the posterior surface (**F**)

**Fig. 3:**
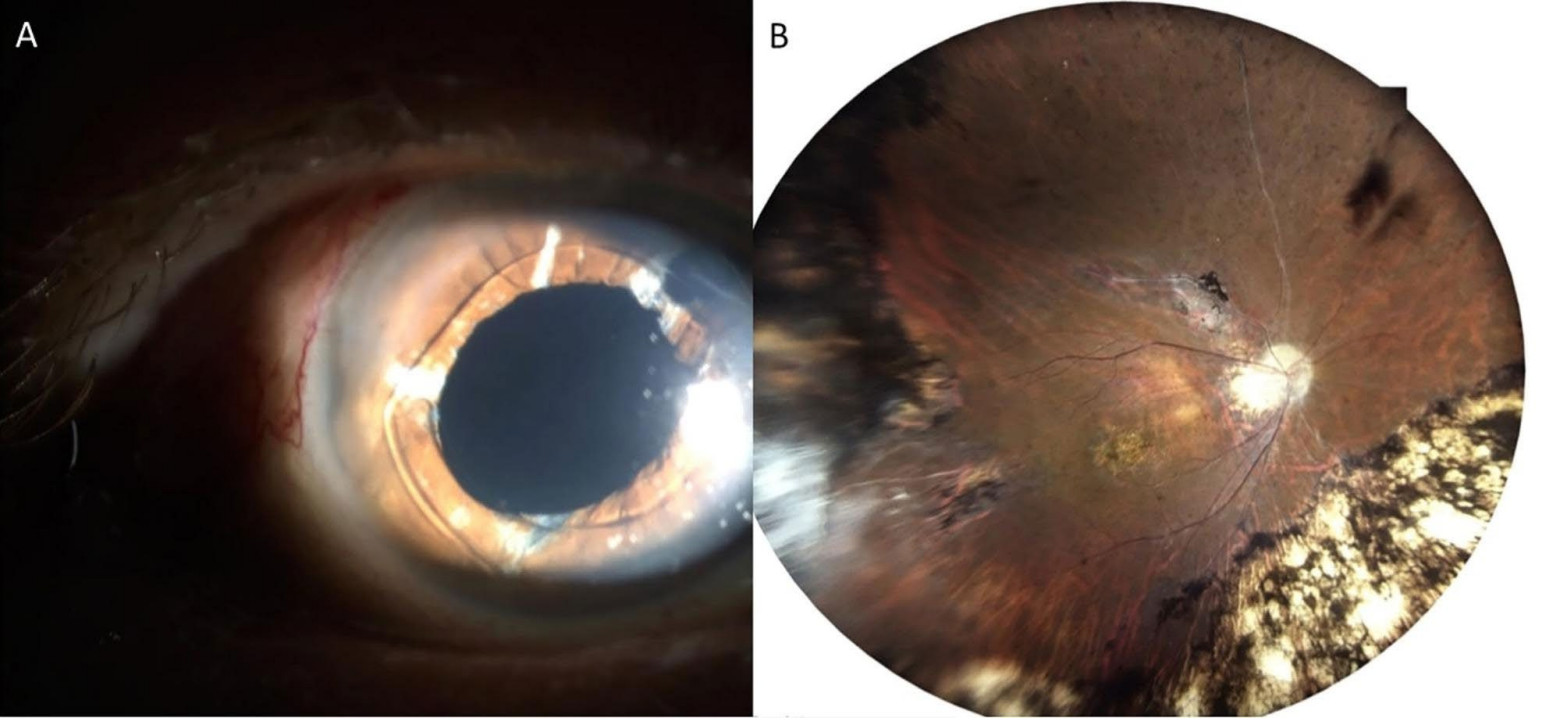
(**A**) Slit-lamp postoperative photograph showing the anterior chamber IOL. (**B**) Postoperative fundus photography by the Optos P200DTx (Optos PLC, Dunfermline, Scotland, UK) camera revealed retinal vascular attenuation, more posterior fundus lesions, and a clearer picture of peripheral pigmented lesions and areas of chorioretinal atrophy compared with the preoperative fundus photograph

An 83-year-old man was referred to our department complaining of vision blurring in his right eye for six months. The patient had a confirmed family history of Wagner syndrome. On examination, best-corrected visual acuity was 20/200 on the right eye and no light perception on the left eye. From his previous ocular history, one year ago he underwent secondary IOL implantation elsewhere with an one-piece foldable hydrophilic acrylic Carlevale IOL (FIL SSF, Soleko IOL Division, Italy) due to IOL dislocation. Slit-lamp examination revealed IOL opacification (Fig. 1A). Cornea and anterior chamber examinations were unremarkable. The results of a noncontact specular microscopy examination showed that his right eye had 1900 cells/mm^2^. Fundus examination by Optos P200DTx (Optos PLC, Dunfermline, Scotland, UK) was difficult due to the opaque lens, but revealed fundus lesions as pigmented condensation, peripheral pigmentary changes and regions of choroidal atrophy (Fig. 1B). After discussing risks and benefits, we proceeded to IOL explantation with a secondary anterior chamber IOL (ACIOL) (MTA4UO; Alcon Laboratories, Inc. Fort Worth, TX 76,134, USA) implantation. After a thorough pars plana vitrectomy, the IOL was removed through a sclero-corneal tunnel incision. However, during the IOL explantation, when the T-shaped haptics “anchors” were withdrawn from the scleral pockets, it was discovered that the IOL was initially implanted upside down, with the anterior surface of the lens facing the vitreous cavity. (Fig. 2A, B, C). The explanted IOL (Fig. 2D, E, F) was immersed in balanced salt solution and was sent for further investigation to laboratory. Three months later, vision improved to 20/25 with no further complications. A slit-lamp examination showed a clear cornea, a quiet anterior chamber, a patent peripheral iridectomy and a well-centered ACIOL (Fig. 3A). Furthermore, corneal endothelial cells were examined and revealed a density of 1730 cells/mm^2^. Postoperative fundus examination revealed vascular attenuation and vascular sheathing with generalized retinal pigment epithelial atrophy with peripheral chorioretinal atrophy (Fig. 3B), identical to the findings of the Wagner syndrome.

### Optical microscopy examination

**Fig. 4:**
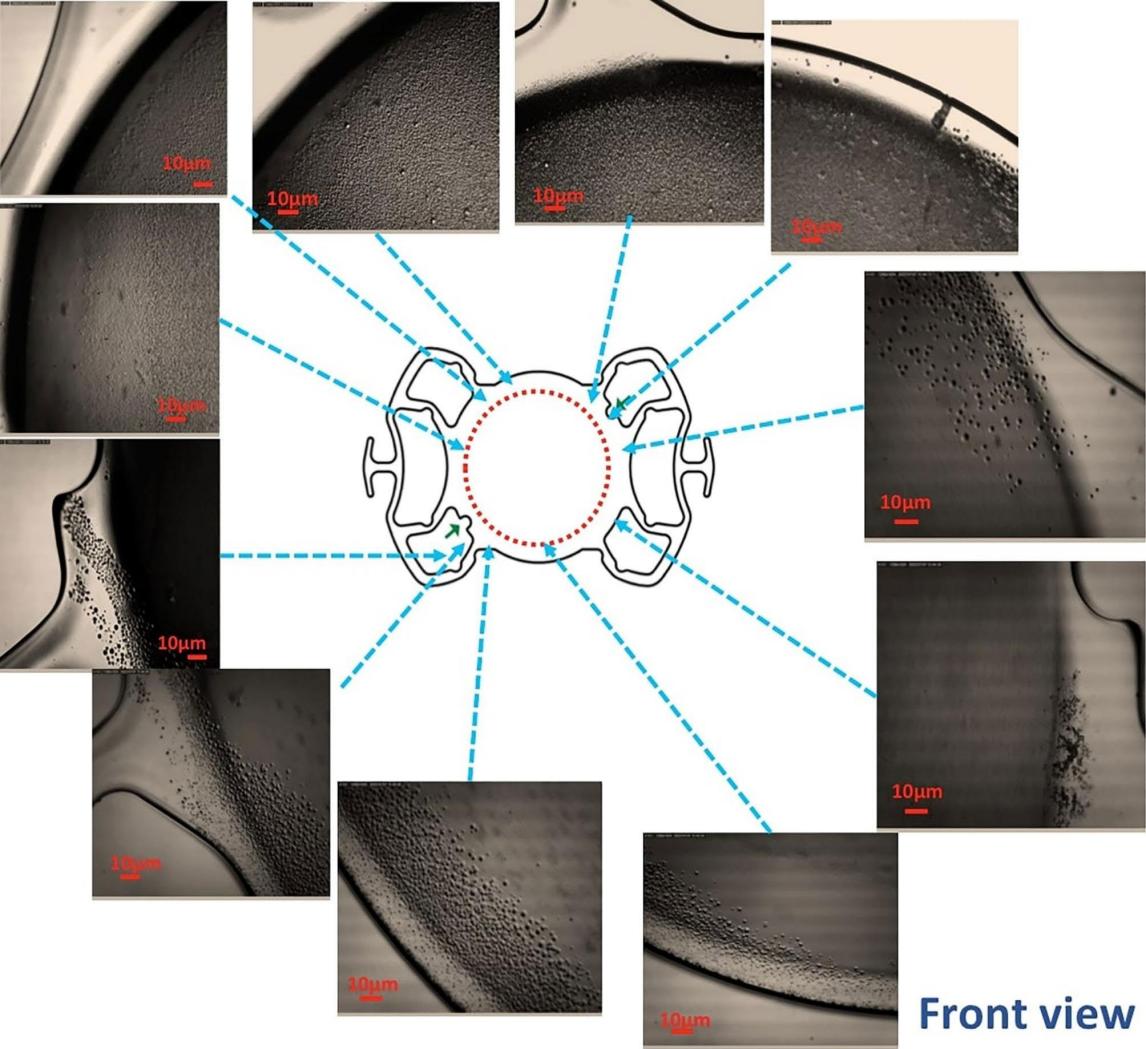
A light microscopy image shows the morphology and particular topographical sedimentation details of the anterior surface of the IOL

**Fig. 5:**
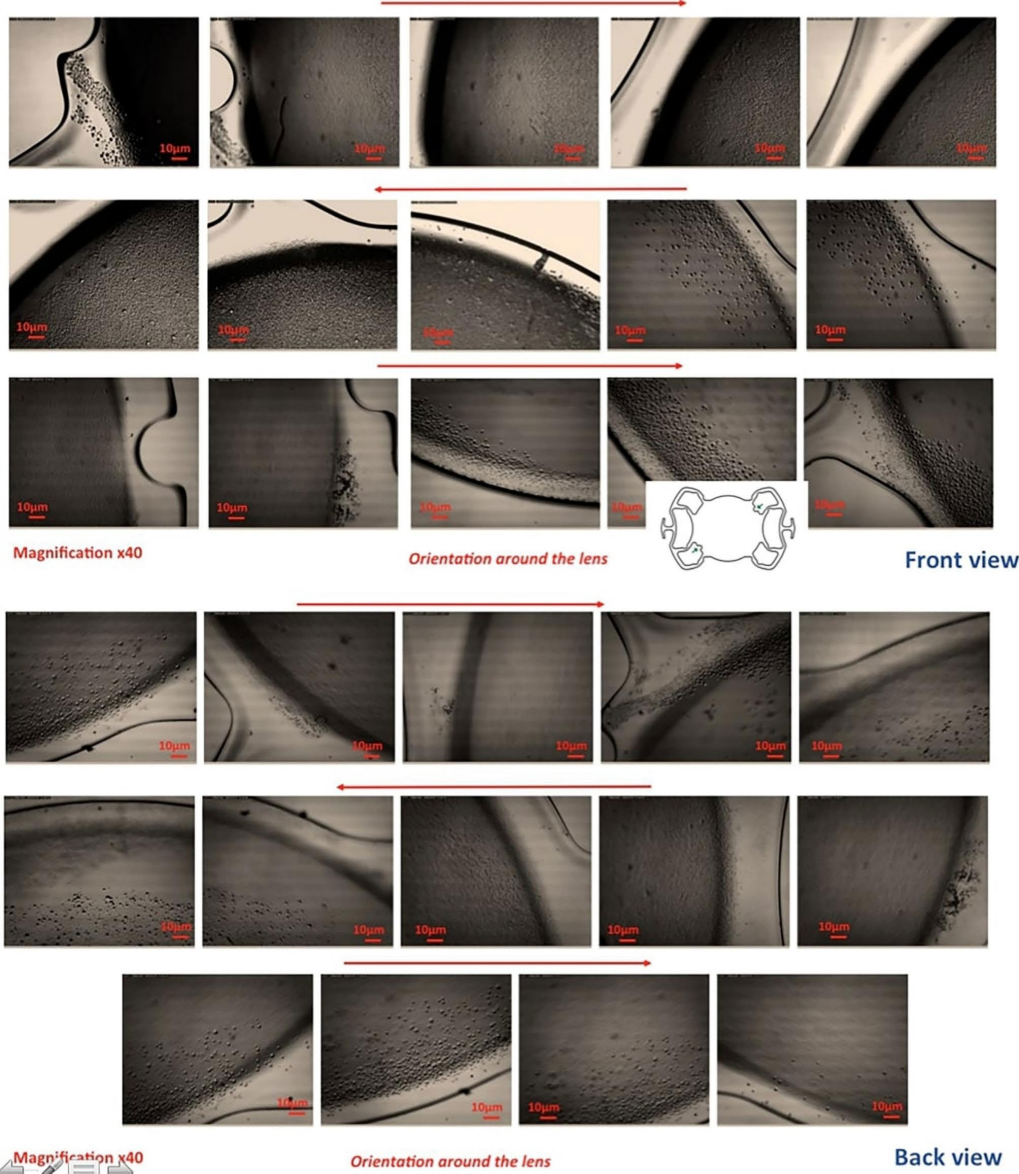
Light microscopy overview images of the anterior surface (Front view) and posterior surface (Back view) of the IOL, as indicated by the scheme, show various degrees of sedimentation characteristics in the peripheral part of the IOL

Optical microscopy IOL examination revealed partial opacification on both surfaces. Under the optical microscope, more deposits peripheral to the lens and a relatively clear center were detected. Detailed examination of the IOL revealed granular opacification more on its anterior surface than on its posterior surface. The morphology and specific real-space topographical opacification details on the anterior and posterior surfaces are indicated in Figs. 4 and 5.

### Scanning Electron Microscopy (SEM) and energy-dispersive x-ray (EDX) spectrometric analysis

After gross examination IOL was dissected and examined by scanning electron microscopy (SEM) equipped with an energy-dispersive X-ray analysis unit (EDX) with a Si detector and a resolution of 129 eV. The IOL was examined on both surfaces following their immobilization on aluminum slabs covered with conductive carbon double-stick tape. Cross sections of the IOL were also examined by securing the cross section in a special split SEM specimen holder.

### Anterior surface

**Fig. 6:**
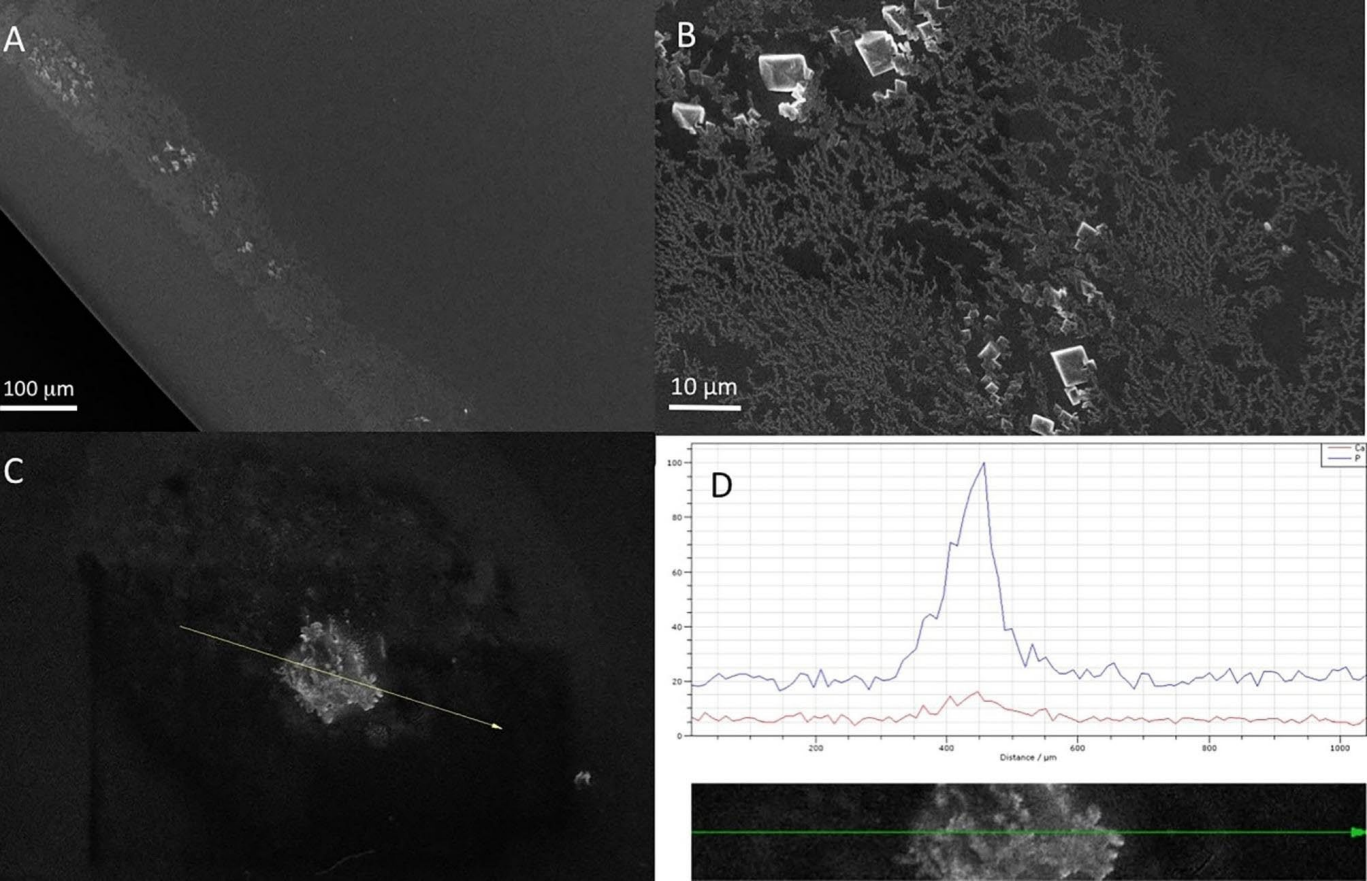
(**A**) Scanning electron microscopy image of the explanted Carlevale IOL (FIL SSF, Soleko IOL Division, Italy) shows a peripheral rim of sodium chloride (from the saline solution) and crystallites of HAP on the anterior surface. (**B**) Scanning electron microscopy image shows a higher magnification of the calcific deposits shown in image A. (**C** and **D**) Energy-dispersive x-ray analysis, including surface formation, exhibiting peaks corresponding to the calcium and phosphorus of the calcific deposits. (**D**) Shows a line-scan elemental microanalysis over the length of the arrow line C. The horizontal axis shows the length scale of microanalysis, and the Y axis shows the relative intensity of the dispersive energy of X-rays emitted by the elements present in the deposit

**Fig. 7:**
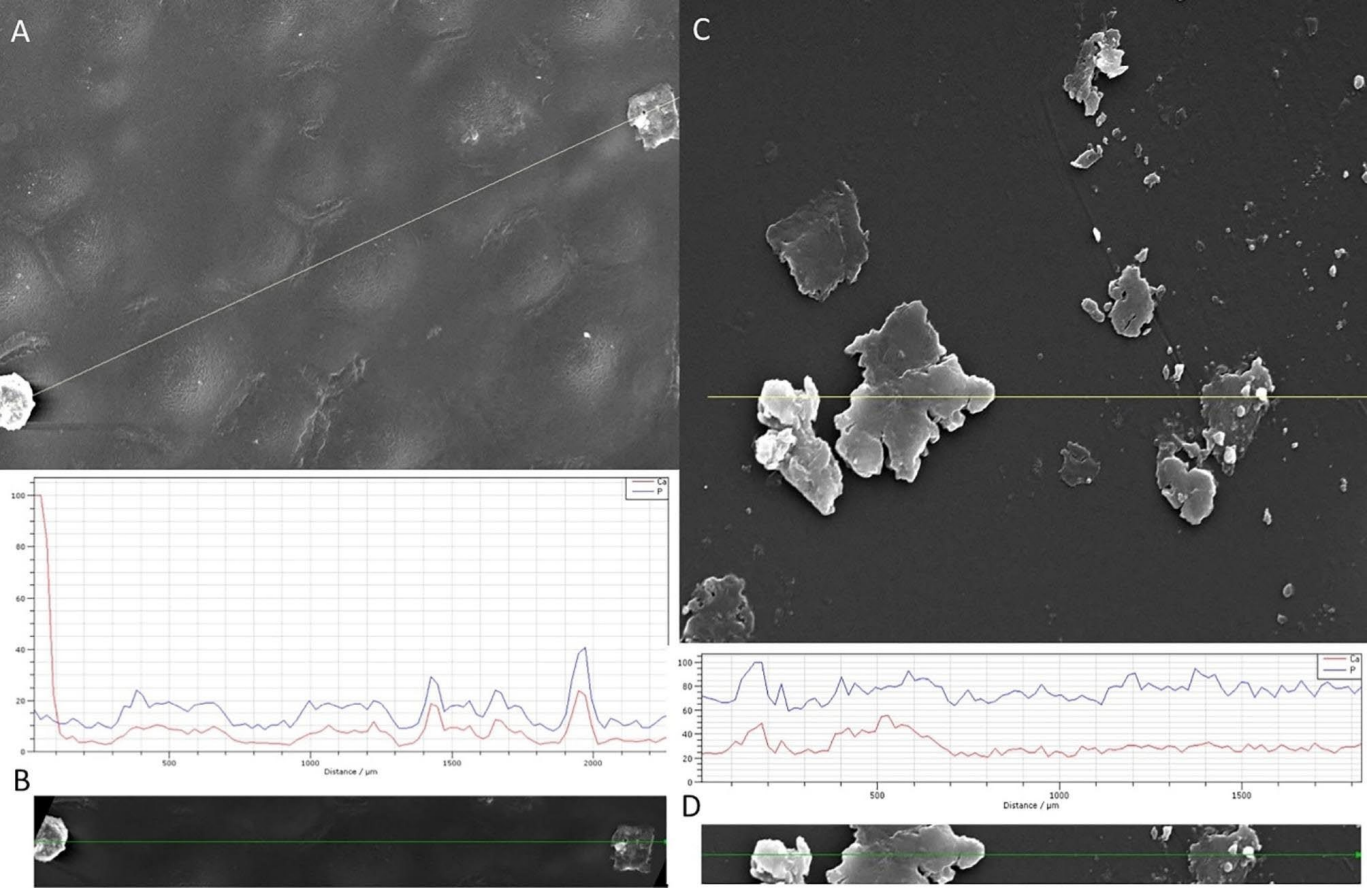
(**A**) Scanning electron microscopy of the anterior surface of the lens reveals a pattern of lumps composed of subsurface calcified deposits as well as surface deposits. The arrow line shows line scan analysis. (**B**) Energy-dispersive x-ray microanalysis, exhibiting peaks corresponding to calcium and phosphorus corresponding to the surface calcium phosphate deposits. In the bottom restricted view of the line scan microanalysis. (**C**) Energy-dispersive x-ray analysis of limited deposits of discrete calcium phosphate crystallites on the posterior surface of the IOL. The arrow line shows the line scan area. (**D**) EDX microanalysis spectrum runs across surface deposits as shown in the bottom

Scanning electron microscopy and EDX microanalysis revealed that IOL had a peripheral rim which consisted mainly of sodium chloride and crystallites of hydroxyapatite (HAP). These deposits, apart from the sodium chloride (lens preservative), consisted of debris of an organic matrix which contained calcium phosphate (Fig. 6). In addition, the anterior surface of the lens showed a surface texture of lumps produced by the underlying calcium phosphate deposits. It should be noted that surface deposits were rather limited (Fig. 7A, B).

### Posterior surface

Surface deposits were significantly reduced in comparison with the corresponding on the anterior surface. The deposits consisted of calcium phosphate embedded in the polymeric or other organic matrix. The morphology of the calcium phosphate crystallites, embedded in the organic matrix, was irregular. There is no clear confirmation of the composition of the deposit particles. Deposits on the posterior surface were very limited and there were no lumps. Furthermore, limited deposits of discrete calcium phosphate crystallites were identified (Fig. 7C, D).

### Overall results

**Fig. 8:**
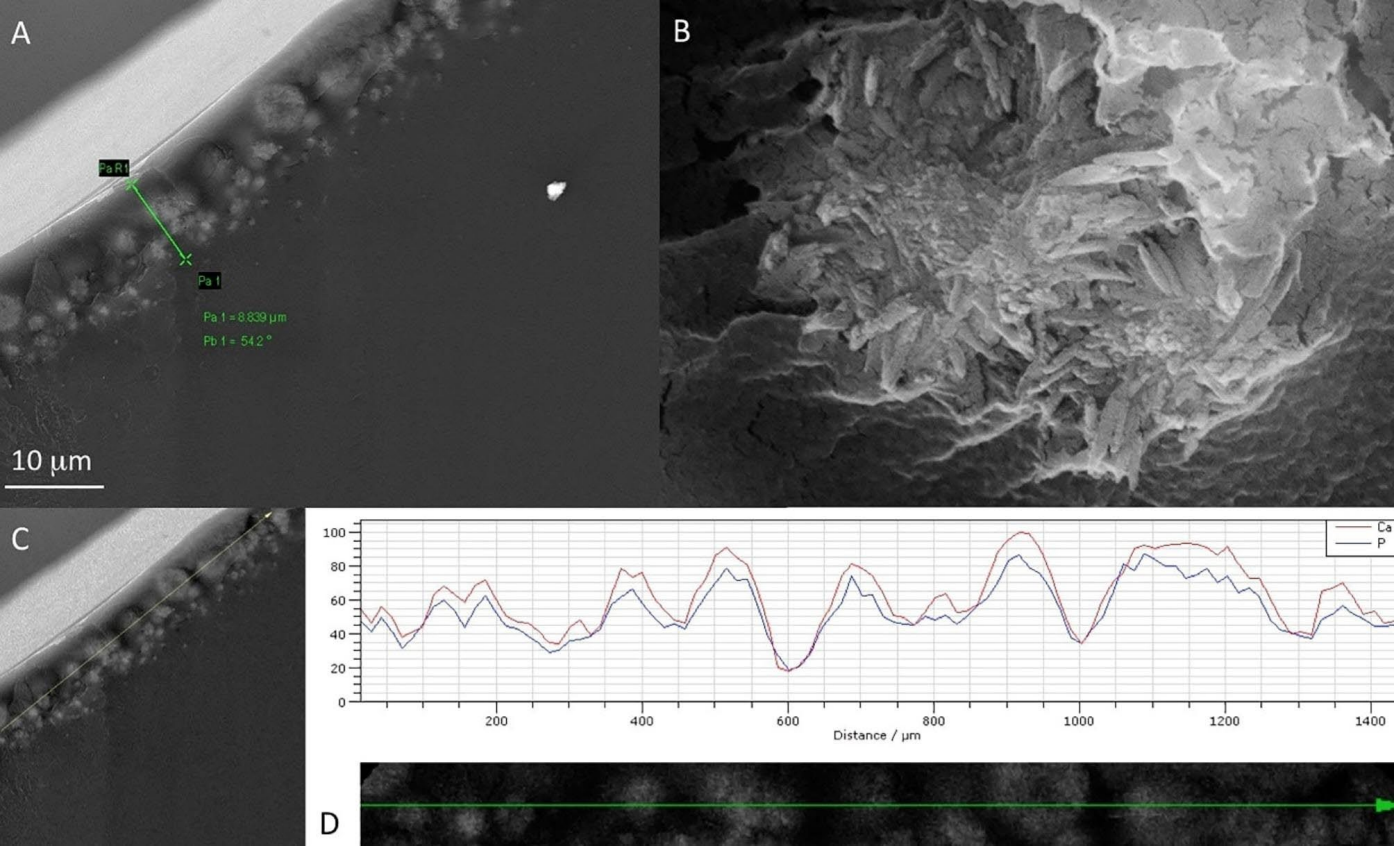
(**A**) SEM image of a cross section of the Carlevale IOL (FIL SSF, Soleko IOL Division, Italy) showing subsurface calcium phosphate deposits forming a dense layer of deposits ca. 10 μm thick in the direction of the anterior external surface towards the interior of the IOL. (**B**) SEM image of the IOL shows the detailed morphology of prismatic crystals of hydroxyapatite formed in the dense layer shown in image A. (**C**) EDX microanalysis of the subsurface deposits (**A**) across the arrow line. (**D**) EDX microanalysis spectrum exhibiting peaks corresponding to calcium and phosphorus in the calcific deposits shown below the spectrum

From the macroscopic IOL examination, opacification was found on the surface of the optical part. Examination under the optical microscope showed more formations peripheral to the lens and a relatively clear center. SEM showed that the anterior surface had significantly more deposits compared to the posterior surface. These deposits, apart from the sodium chloride (lens preservative), consisted of an organic matrix containing calcium phosphate. On the posterior surface, the deposits were similar, although no sodium chloride was observed, which on the anterior surface appeared to be strongly retained and was not removed despite extensive washing of the IOL with triply distilled water. IOL cross section examination showed the presence of a layer of aggregates of prismatic nanocrystals of hydroxyapatite (HAP) below the anterior surface. Penetration depth of HAP formations reached up to a depth of 60 μm from the posterior surface (penetration of about 550 μm from the anterior surface). The macroscopically observed opacification is due to the formation of crystals clusters below the anterior surface, forming a dense layer about 10 μm thick. The calcific deposits were very limited at greater depths (Fig. 8). There was no layer of HAP crystals under the posterior surface. Despite the weakness of the signals, which is due to the very small amounts of the salt in the specific location, the identification of the formations as calcium phosphate was unambiguous. The IOL opacification was due to HAP formation by diffusion of the crystal lattice ions within the hydrophilic polymer.

## Discussion and conclusions

Carlevale IOL (FIL SSF, Soleko IOL Division, Italy) has gained popularity among surgeons by offering several advantages in cases requiring scleral fixation IOLs. Several authors have observed decreased surgical complexity and duration of surgery, minimal incidence of IOL tilt, and strong attachment of the T-shaped plugs to the sclera, which assure IOL stability [1, 9−10] Furthermore, IOL haptics have 10° anterior angulation with respect to the optic plate in order to ensure a more physiologic and effective lens placement, minimizing pupillary block likelihood, and preventing iris rubbing. Our study highlights for the first time a case of a sutureless Carlevale lens (FIL SSF, Soleko IOL Division, Italy) that underwent calcification after secondary implantation due to dislocation via pars plana vitrectomy. Opacification in our case was permanent contrary to earlier report of temporary opacification ascribed to temperature differences [11] In hydrophilic IOLs opacification is due to calcification catalyzed by the functional groups (-OH and/or –COOH) on the surface of IOL made of Poly(hydroxyethyl) methacrylate (PHEMA) playing the most important role [2–5, 12].

In our case, the patient began to experience significant glare, which gradually evolved into diminished vision, necessitating IOL explantation. During surgical procedure, reverse IOL implantation was demonstrated as shown in Fig. 1B. It is very likely that reverse position may favor complications as the posterior iris epithelium rubs on the posterior surface of the IOL due to abnormal lens placement. Regarding the mechanism of IOL calcification, the following two hypotheses are suggested. First, a possible mechanical rubbing of the posterior iris epithelium on the IOL can promote the breakdown of the blood-aqueous barrier. Second, Wagner syndrome as a hereditary vitreoretinal degeneration disorder may also be associated with a disruption of the blood-retinal barrier [13] Consequently, there is a generalized disruption of the blood-ocular barrier, permitting the leakage of serum proteins and inorganic ions into the anterior and posterior chambers, as well as into the vitreous cavity which increase the thermodynamic driving force for calcification. According to earlier research, diseases and ophthalmic procedures are associated with the breakdown of the blood-aqueous barrier, which may be a major contributing factor in promoting IOL calcification [3,14] The diversification of calcification, i.e. the quantitative difference of calcific deposits between the anterior and posterior surfaces, could be attributed to the dispersion of inorganic components mainly into the vitreous cavity. As disruption of the blood-ocular barrier is a risk factor for hydrophilic IOL calcification, an MTA4UO (Alcon Laboratories, Inc. Fort Worth, TX 76,134, USA) intraocular lens made of PMMA, which is a hydrophobic material, was implanted. From the existing literature, there has been no demonstrated calcification in hydrophobic material. We chose an ACIOL because the patient had no prior history of glaucoma, and furthermore, anterior chamber lenses are effective in treating aphakia in vitrectomized eyes [15].

The findings in our case, however, refer to the hydrophilic Carlevale IOL (FIL SSF, Soleko IOL Division, Italy), whereas there is currently no data available about opacification/calcification regarding the high-tech Carlevale IOL model (md Tech, Casoria (NA), Italy) that is manufactured by a hydrophilic-hydrophobic copolymer.

In conclusion, it was shown that the macroscopically observed opacification of the IOL was due to the formation of hydroxyapatite (Ca_5_(PO_4_)_3_OH, HAP) crystals below the anterior surface of the IOL, which formed a dense layer ca. 10 μm thick. Calcification was due to HAP formation by diffusion of the HAP lattice ions (Ca^2+,^ PO_4_^3−^ and OH-) within the hydrophilic polymer. Penetration depth for HAP formation reached 60 μm from the posterior surface. There was no layer of HAP crystals under the posterior surface of the IOL. In fact, the reverse position of the IOL may be related to a possible prolonged rubbing of the posterior iris epithelium on the IOL, resulting in a disruption of the blood-ocular barrier. To the best of our knowledge, this is the first case reporting laboratory evidence of calcification of a Carlevale IOL (FIL SSF, Soleko IOL Division, Italy). Long-term postoperative surveillance is needed following Carlevale IOL (FIL SSF, Soleko IOL Division, Italy) implantation in order to determine the real incidence of this clinical entity.

### Electronic supplementary material

Below is the link to the electronic supplementary material.

Supplementary Material 1

## Data Availability

Data is available from the corresponding author on reasonable request.

## Abbreviations

IOL: Intraocular lens
SEM: Scanning electron microscopy
EDX: Energy-dispersive x-ray spectroscopy
SSF: Sutureless scleral fixation
ACIOL: Anterior chamber intraocular lens
HAP: Hydroxyapatite
PHEMA: Poly-hydroxyethyl-methacrylate

## References

[1] Rossi T, Iannetta D, Romano V, Carlevale C, Forlini M, Telani S (2021). A novel intraocular lens designed for sutureless scleral fixation: surgical series. Graefes Arch Clin Exp Ophthalmol.

[2] Schrittenlocher S, Schaub F, Hos D, Siebelmann S, Cursiefen C, Bachmann B (2018). Evolution of Consecutive Descemet membrane endothelial keratoplasty outcomes throughout a 5-Year period performed by two experienced Surgeons. Am J Ophthalmol.

[3] Nakanome S, Watanabe H, Tanaka K, Tochikubo T (2008). Calcification of Hydroview H60M intraocular lenses: aqueous humor analysis and comparisons with other intraocular lens materials. J Cataract Refract Surg.

[4] Werner L (2008). Calcification of hydrophilic acrylic intraocular lenses. Am J Ophthalmol.

[5] Drimtzias EG, Rokidi SG, Gartaganis SP, Koutsoukos PG. Experimental investigation on mechanism of hydrophilic acrylic intraocular lens calcification. Am J Ophthalmol. 2011;152. 10.1016/j.ajo.2011.04.009. :824 – 33.e1. 10.1016/j.ajo.2011.04.009 21763638

[6] Georgalas I, Spyropoulos D, Gotzaridis S, Papakonstantinou E, Kandarakis S, Kanakis M (2022). Scleral fixation of Carlevale intraocular lens: a new tool in correcting aphakia with no capsular support. Eur J Ophthalmol.

[7] Izak A, Werner L, Pandey S, Apple DJ (2003). Calcification of modern foldable hydrogel intraocular lens designs. Eye.

[8] Gartaganis SP, Kanellopoulou DG, Mela EK, Panteli VS, Koutsoukos PG (2008). Opacification of hydrophilic acrylic intraocular lens attributable to calcification: investigation on mechanism. Am J Ophthalmol.

[9] Veronese C, Maiolo C, Armstrong GW, Primavera L, Torrazza C, Della Mora L (2020). New surgical approach for sutureless scleral fixation. Eur J Ophthalmol.

[10] Barca F, Caporossi T, de Angelis L, Giansanti F, Savastano A, Di Leo L (2020). Trans-scleral plugs fixated IOL: a new paradigm for sutureless scleral fixation. J Cataract Refract Surg.

[11] Danese C, Calabresi R, Lanzetta P (2021). Transient clouding of a Sutureless Scleral Fixated hydrophilic intraocular Lens with spontaneous resolution: a Case Report and in vitro experimental test. Case Rep Ophthalmol.

[12] Koutsoukos PG, Natsi PD, Gartaganis PS, Gartaganis SP. Calcification of biomaterials, In Water-Formed Deposits. Fundamentals and mitigation strategies, Amjad Z, Dermadis K, Eds E. 2022 pp. 495–509. 10.1016/B978-0-12-822896-8.00019-4.

[13] Strong S, Liew G, Michaelides M (2017). Retinitis pigmentosa-associated cystoid macular oedema: pathogenesis and avenues of intervention. Br J Ophthalmol.

[14] Neuhann IM, Kleinmann G, Apple DJ (2008). A new classification of calcification of intraocular lenses. Ophthalmology.

[15] Negretti GS, Lai M, Petrou P, Walker R, Charteris D (2018). Anterior chamber lens implantation in vitrectomised eyes. Eye (Lond).

